# Shema Oral Liquid Ameliorates the Severity of LPS-Induced COPD *via* Regulating DNMT1

**DOI:** 10.3389/fphar.2022.903593

**Published:** 2022-06-08

**Authors:** Fangbo Zhang, Feifei Guo, Yang Liu, Yi Zhang, Defeng Li, Hongjun Yang

**Affiliations:** ^1^ Institute of Chinese Materia Medica, China Academy of Chinese Medical Sciences, Beijing, China; ^2^ Experimental Research Center, China Academy of Chinese Medical Sciences, Beijing, China

**Keywords:** chronic obstructive pulmonary disease, shema oral liquid, network pharmacology, quantitative proteomics, lipopolysaccharide, DNMT1

## Abstract

**Background:** Chronic obstructive pulmonary disease (COPD) is the most common respiratory disease with high morbidity and mortality. Shema oral liquid (Shema) is a traditional Chinese medicine (TCM) approved for the treatment of respiratory diseases. Clinical applications have shown that Shema has antitussive, expectorant, and anti-asthmatic effects, but its definite efficacy to COPD is still unclear. This study aimed to explore the therapeutic capacity and potential mechanism of Shema in treatment of COPD.

**Methods:** Network pharmacology was used to investigated the possible pharmacological mechanism of Shema against COPD. A rat model of lipopolysaccharide (LPS)-induced COPD was established to determine pulmonary ventilatory function, serum inflammatory cytokines, and pulmonary pathological change. Subsequently, tandem mass tag (TMT)-based quantitative proteomics was used to further reveal the therapeutic targets related with Shema against COPD. Western blot was finally performed to validate the expression of targeted proteins screened by proteomics research.

**Results:** Network pharmacology analysis indicated that Shema against COPD mainly inhibited the inflammation and affected the immune system. The animal experiment demonstrated that Shema treatment protected the lung tissue from LPS induced injury, inhibited the levels of serum inflammatory cytokines such as interleukin (IL)-1β, IL-6, IL-8, and tumor necrosis factor (TNF)-α, and improved the respiratory ventilatory function by upregulating forced expiratory volume in 0.1 s (FEV0.1), FEV0.3, forced vital capacity (FVC), and the ratios of FEV0.1 (0.3)/FVC. Proteomic analysis and western blot both proved that Shema inhibited the expression of DNA methyltransferase 1 (DNMT1) in the lung tissue.

**Conclusion:** The therapeutic mechanism of Shema in treatment of COPD may involve inhibiting inflammatory response, improving pulmonary ventilatory function, and alleviating LPS-induced lung injury through regulating the expression of DNMT1. This study also shed light on the development of therapeutic strategies in treating COPD by intervening DNMT-related pathways.

## Introduction

Chronic obstructive pulmonary disease (COPD) is a prevalent and chronic respiratory disease characterized by expiratory airflow limitation, chronic systemic inflammation, airway remodeling and lung emphysema, leading to a progressive decline in pulmonary function (Wang et al., 2018). COPD is a severe global public health challenge, and it is also one of the leading causes of death worldwide (Halpin et al., 2017). The pathogenesis of COPD includes inflammatory reaction (Ghobadi et al., 2017), protease-antiprotease imbalance (Mohan et al., 2016), oxidative stress (Hikichi et al., 2019), muscle dysfunction and lung microbiome (Huang et al., 2014; Barreiro and Jaitovich, 2018). Several types of drugs such as antibiotics, steroids, immunomodulators, antitussives, expectorants, bronchodilators, and leukotriene receptor antagonists are frequently used to suppress the symptoms of COPD, but they have limited clinical efficacy (Jeffery, 2001). Thus, deeper researches focusing on increasing our understanding of pathogenic mechanism and developing novel therapies are urgently needed.

Traditional Chinese medicine (TCM) has been widely used for centuries in China. Their proven efficacy, minimal side effects and low cost are considered valuable as alternative or complementary medicines for health care and disease treatment worldwide. A traditional Chinese prescription Shema oral liquid (Shema) comes from the records including “Shegan Mahuang Decoction” in the Synopsis of Golden Chamber and “Maxing Shigan Decoction” in the Treatise on Febrile Diseases. The formula consists of ten types of TCMs: *Ephedra Herba* (Ma-Huang), *Arisaematis Rhizoma* (Dan-Nan-Xing, mixed with oxgall), *Morus alba* L. (Sang-Bai-Pi), *Belamcandae Rhizome* (She-Gan), *Raphani Semen* (Lai-Fu-Zi), *Amygdalus Communis* Vas (Xing-Ren), *Cynanchum glaucescems* (Bai-Qian), *Scutellariae Radix* (Huang-Qin), *Schisandrae Chinensis Fructus* (Wu-Wei-Zi), and Gypsum Fibrosum (Shi-Gao), which has been approved for the treatment of respiratory diseases. Clinical applications have indicated that Shema has antitussive, expectorant, and anti-asthmatic effects (Cheng, 2021). However, the precise efficacy and mechanism of Shema for the treatment of COPD is unclear.

Here, we used network pharmacology to predict the possible pharmacological mechanism of Shema in treatment of COPD, which could provide the theoretical basis for the mechanistic study. Next, we established a lipopolysaccharide (LPS)-induced COPD rat model and confirmed the molecular targets concluded from the network pharmacology analysis. Then quantitative proteomics and western blot were performed to further illustrate the potential therapeutic targets of Shema against COPD. Therefore, our study provided the definite investigation of clarifying the efficacy and mechanism of Shema against COPD.

## Materials and Methods

### Network Pharmacology Analysis of Shema in Treatment of COPD

The chemical ingredients of Shema were obtained from TCMID database (http://www.megabionet.org/tcmid/) (Xue et al., 2012). The molecular targets of ingredients from Shema were mainly collected from BATMAN-TCM database (http://bionet.ncpsb.org/batman-tcm/). The major source of COPD-related disease targets was acquired from two databases including Human Phenotype Ontology (HPO, https://hpo.jax.org/app/) and DisGeNET (http://www.disgenet.org/). The potential targets of Shema and the disease targets of COPD were combined and imported to STRING database (http://www.string-db.org/) to generate a protein-protein interaction (PPI) network (Szklarczyk et al., 2017). The PPI network was created by Cytoscape software (version 3.7.2, Boston, MA, United States). In order to confirm the possible pharmacologic effect of Shema in treatment of COPD, David database (version 6.8, https://david.ncifcrf.gov/) was used to perform Gene Ontology (GO) and Kyoto Encyclopedia of genes and Genomes (KEGG) enrichment analysis.

### Experimental Animals

Male Sprague-Dawley rats (180–220 g) were obtained from Beijing HFK Bioscience Co. Ltd., China (license number was SCXK (Jing) 2016-0004). All experiments were designed and handled in accordance with the local ethical guidelines for animal care and usage. Before the experiment, rats were adapted to the feeding environment for 3 days.

### Drugs

Shema is composed of ten different TCMs as followings: Ma-Huang, Dan-Nan-Xing, Sang-Bai-Pi, She-Gan, Lai-Fu-Zi, Xing-Ren, Bai-Qian, Huang-Qin, Wu-Wei-Zi, and Shi-Gao (weight ratio 3: 3: 5: 5: 4: 5: 5: 5: 3: 10). The herbal extract was prepared by Hainan Zhongshenghemei Biopharmaceutical Co. Ltd., China. The extract was dissolved in distilled water. The clinical oral dose of Shema was 0.5 ml/kg/day (60 kg, 10 ml/time, t.i.d.), which was equivalent to the gavage dose of 3.125 ml/kg/day for the rats (200 g). Therefore, we set 1.0, 3.0, and 6.0 ml/kg/day as the gavage doses in our research. Dexamethasone tablets (0.75 mg/Tablet) were obtained from Guangdong South Land Pharmaceutical Co. Ltd., China.

### COPD Animal Model Construction

The rat model of COPD was constructed as previously reported (Vernooy et al., 2002). The rats were anesthetized with 1% pentobarbital sodium (40 mg/kg, Sigma, MA, United States) intraperitoneally, followed by a tracheal injection of 0.2 ml LPS (1.0 μg/μL, in 0.9% saline, from *Escherichia coli* 055: B5, solarbio, Beijing, China) through an endotracheal tube (rat 14G type, Zhongyanboji, Beijing, China) twice a week. According to the gavage doses, the rats were randomly divided into six groups (*n* = 12 per group): control group (Control), COPD model group (COPD), Shema low-dose group (Shema 1.0 ml/kg), Shema medium-dose group (Shema 3.0 ml/kg), Shema high-dose group (Shema 6.0 ml/kg), and dexamethasone group (DXMS 2.0 mg/kg). The rats in Shema or DXMS treated groups were administered intragastrically with Shema or dexamethasone once daily for 21 days continuously. The rats in the control group were injected with the same volume of saline twice a week, and administered with distilled water for 21 days. On the 22nd day, we monitored the pulmonary ventilatory function of the rats, and then sacrificed them to do various experiments.

### Pulmonary Ventilatory Function Measurement

After intraperitoneal anesthesia by 1% pentobarbital sodium (40 mg/kg), six rats from all groups were randomly selected for the measurement of pulmonary ventilatory function. The subcutaneous tissue was separated to expose the trachea, and then endotracheal intubation was performed. The rat was connected with a pulmonary functionality test system (AniRes2005 analytic system, Beijing Bestlab High-Tech, China) to measure forced expiratory volume in 0.1 s (FEV0.1), forced expiratory volume in 0.3 s (FEV0.3) and forced vital capacity (FVC).

### Pulmonary Histopathologic Examination

The left lung was removed immediately and fixed in 4% paraformaldehyde (Solarbio, Beijing, China). The tissue was embedded in paraffin, cut into 5 μm thick slices, and then dyed with hematoxylin-eosin (HE). The histological change of the slide was observed under the light microscopy (Olympus, Tokyo, Japan). Five random photographs of each slide were obtained. The histological score was evaluated *via* the following indicators: 1) pulmonary emphysema; 2) alveolar edema; 3) inflammatory cell infiltration; 4) alveolar hemorrhage; 5) destroyed alveolar. Each item was scored as follows: 0 for normal, 1 for slight, 2 for moderate, and 3 for severe (Sun et al., 2011). The accumulated scores were regarded as the pulmonary histological score of the sample.

### Serum Inflammatory Cytokines Detection

Peripheral blood was obtained from the retro-orbital venous plexus on the 11th and 21st day, respectively, followed by the centrifugation to collect the supernatant. The serum levels of interleukin (IL)-1β, IL-6, IL-13, and tumor necrosis factor (TNF)-α were detected by enzyme-linked immunosorbent assay (ELISA) kits (Huamei, Wuhan, China). All the procedures were completed according to the strict instruction of the manufacturer.

### TMT-Based Quantitative Proteomic Analysis

Nine biological replicates (*n* = 3/group) were identified from the control group, COPD group, and Shema 6.0 ml/kg group. Proteins from the middle lobe of the right lung tissue were extracted by lysis solution (containing 2% SDS and 7 M urea) mixed with 1× protease inhibitor cocktail (Sigma, MA, United States). The protein sample was centrifuged at 13,000 g for 15 min at 4°C, and the supernatant was gained. The BCA protein assay kit (Pierce, CA, United States) was used to measure the concentration of the protein sample. After a series of treatments, the sample was finally labeled with a TMT10 plex Isobaric Label Reagent Set (Thermo Scientific, MA, United States). The TMT-labeled peptide sample was separated using the Ultimate 3000 HPLC operating system (Thermo Scientific, MA, United States). The fractionated samples were centrifuged, condensed, and then dried by a vacuum concentrator. Subsequently, the samples were redissolved in 0.1% formic acid (in aqueous solution) and analyzed with an EASY-nLC1200 Orbitrap Elite (Thermo Scientific, CA, United States). The differentially expressed proteins (DEPs) were regarded as significantly expressed proteins based on fold change >1.20 or <0.80 as well as *p*-value < 0.05.

### Western Blot

Tissue lysate from the middle lobe of the right lung tissue was prepared using ice-cold RIPA lysis buffer and protease inhibitors (Solarbio, Beijing, China). Samples were crushed by the ultrasonic wave (Xinzhi, Ningbo, China), and then centrifuged at 13,000 g for 15 min (4°C). The concentration of each protein sample was determined by a BCA protein assay kit (Pierce, CA, United States). Next, 40 μg protein was separated through sodium dodecyl sulfate-polyacrylamide gel electrophoresis, and transferred onto a PVDF membrane (Millipore, Darmstadt, Germany). The membrane was blocked with a diluted primary antibody DNMT1 (1:1000, cat. no. 5032, Cell Signaling Technology, MA, United States) overnight at 4°C. *β*-actin (1:1000, cat. no. 4970, Cell Signaling Technology, MA, United States) served as an internal reference. After incubation with a secondary antibody, the protein band was examined with an enhanced chemiluminescence agent (Millipore, MA, United States).

### Statistical Analysis

SPSS 20.0 software was used for statistical analysis. Data were presented as mean ± standard deviation (SD). One-way analysis of variance (ANOVA) and Student’s *t*-test were used for comparisons. In proteomics research Scaffold software was employed for data analysis, and Mann-Whitney test was used to compare differences between two groups. Values with *p* < 0.05 were accepted as statistically significant.

## Results

### PPI Network Construction and Functional Enrichment Analysis of Shema Against COPD

**FIGURE 1:**
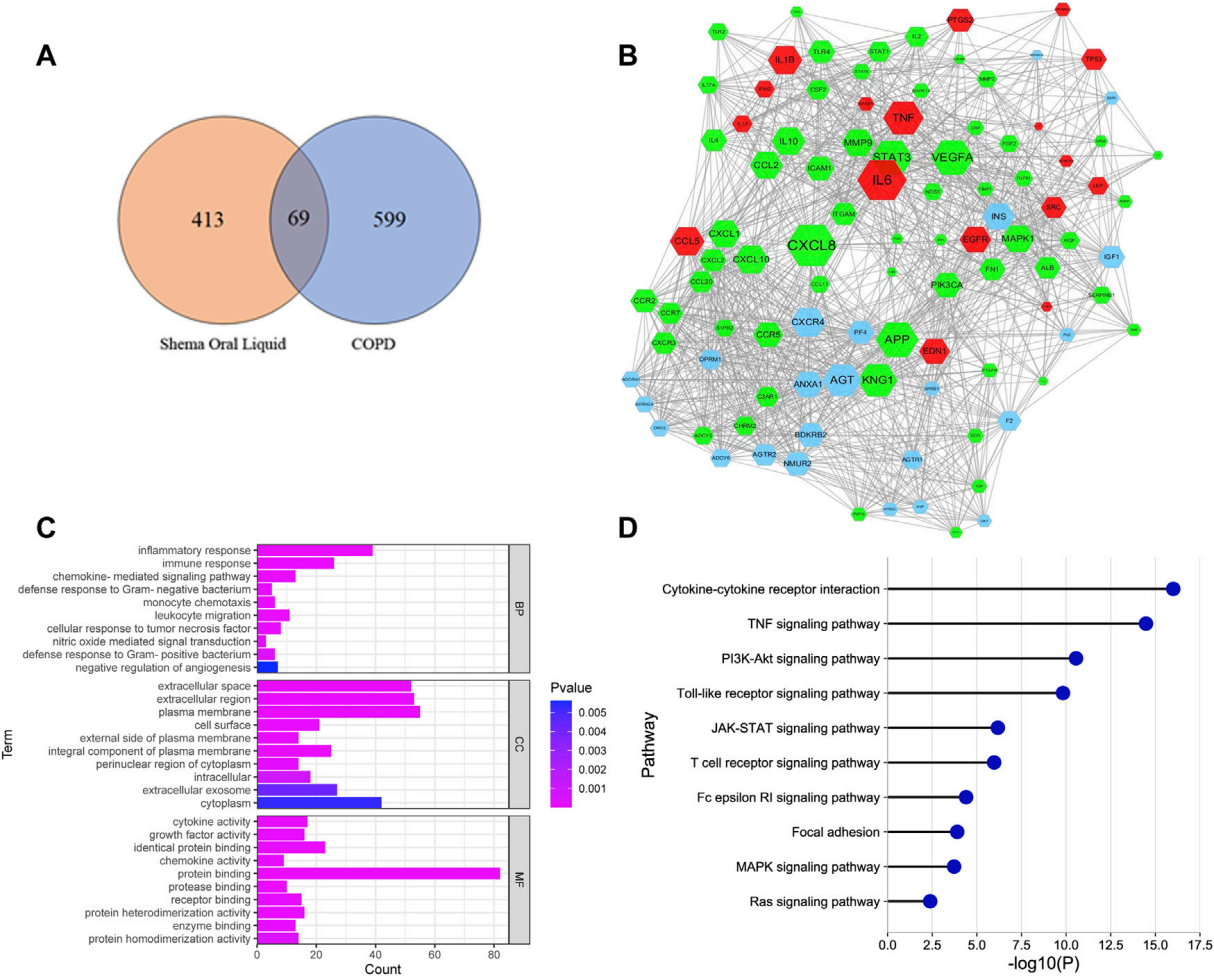
Network pharmacology analysis of Shema in treatment of COPD. **(A)** Venn diagram between the drug targets of Shema and the disease targets of COPD. **(B)** PPI network constructed on the basis of Shema targets and COPD targets. Blue hexagon nodes represented the targets of Shema. Green hexagon nodes represented the targets of COPD. Red hexagon nodes represented the overlapping targets between Shema and COPD. The interaction with confidence score >0.8 was selected. **(C)** GO enrichment analysis of the candidate targets for Shema against COPD including BP, CC and MF terms. **(D)** KEGG pathway enrichment analysis of the candidate targets for Shema against COPD.

355 chemical compounds and 482 putative targets of Shema were obtained, and 668 molecular targets of COPD were collected (Supplementary Table S1). There were 69 overlapping genes between Shema targets and COPD targets (Figure 1A, Supplementary Table S2). The potential targets of Shema and the disease targets of COPD were uploaded to STRING database to construct a PPI network, and protein interaction data with high confidence of a score >0.8 was selected (Figure 1B). Figure 1B showed that inflammatory cytokines such as IL-1β, IL-6, IL-13, and TNF-α were centrally located in the PPI network, suggesting that these molecular targets may be related with the pathogenesis of COPD and the treatment of Shema.

GO analysis showed that the most significantly enriched categories in biology process (BP) term were correlated with chemokine mediated signaling pathway, nitric oxide-mediated signal transduction, monocyte chemotaxis, negative regulation of angiogenesis, inflammatory response, cellular response to tumor necrosis factor, immune response, and defense response to Gram-positive/negative bacterium (Figure 1C). KEGG analysis showed that the signaling pathways involved in Shema against COPD were mainly associated with cytokine-cytokine receptor interaction, focal adhesion, Ras, TNF, MAPK, PI3K-Akt, JAK-STAT, T cell receptor, Toll-like receptor, and Fc epsilon RI signaling pathways (Figure 1D).

### Shema Enhanced the Pulmonary Ventilatory Functionality

**FIGURE 2:**
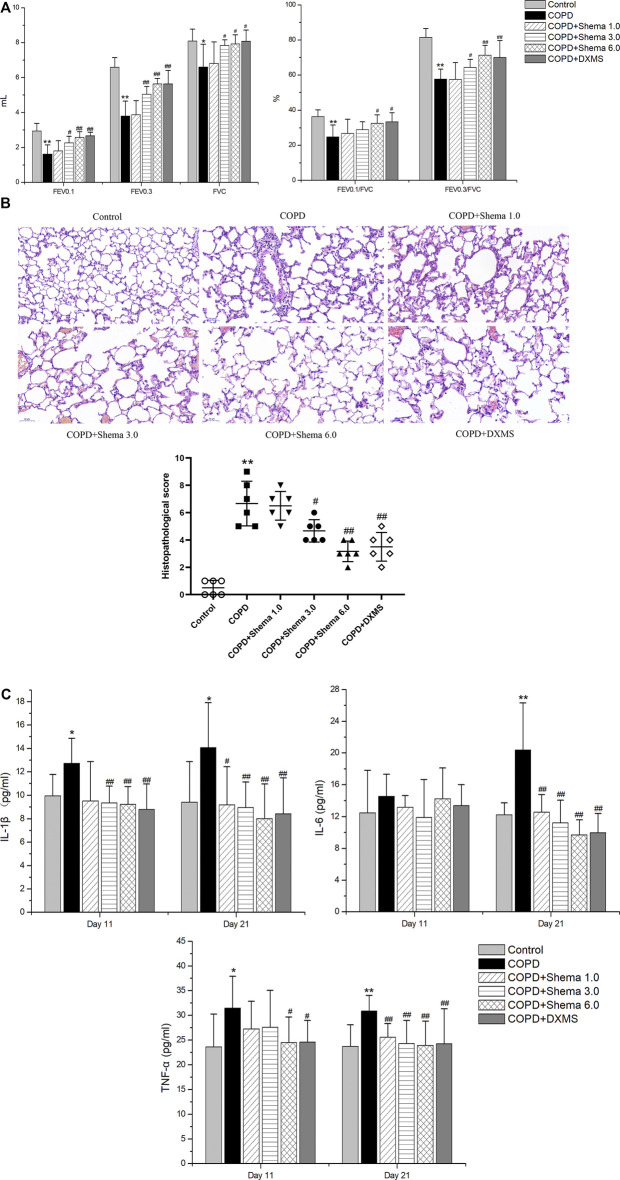
(Continued).

The parameters involving FEV0.1, FEV0.3, and FVC were measured to observe the impairment of pulmonary ventilatory function after Shema treatment. The values of FEV0.1, FEV0.3, and FVC in the COPD group were significantly decreased compared with the control group, while these values in Shema 3.0 and 6.0 groups were significantly elevated compared with the COPD group. Moreover, the ratios of FEV0.1/FVC and FEV0.3/FVC in the COPD group were significantly downregulated compared with the control group, but these two ratios in Shema 6.0 group were significantly increased compared with the COPD group (Figure 2A).

### Shema Alleviated the Lung Tissue Injury Induced by LPS

The structures of the bronchiole and pulmonary alveolus were normal in the control group. However, the alveolar wall and septum was thickened, the alveolar structure was even disordered, extensive inflammatory cells were infiltrated in the airway wall, and pulmonary edema was observed in the COPD group. The inflammatory cell infiltration was decreased, and the alveolar fluid exudation was reduced in the Shema 3.0 and 6.0 groups. The histological score in the COPD group was significantly increased compared with the control group. Meanwhile, the histopathological scores in the Shema 3.0 and 6.0 groups were dramatically declined compared with the COPD group (Figure 2B).

### Shema Reduced the Levels of Serum Inflammatory Cytokines

To identify whether the inflammatory cytokines concluded from network pharmacology results were involved in the therapeutic targets of Shema treatment, these cytokines in the serum were detected by ELISA test. Compared with the control group, the levels of IL-1β and TNF-α in the COPD group were markedly increased on the 11th day, and the levels of IL-1β, IL-6, and TNF-α in the COPD group were increased on the 21st day. Compared with the COPD group, the level of TNF-α in the Shema 6.0 group was decreased, and the levels of IL-1β in both Shema 3.0 and 6.0 group were decreased on the 11th day; the levels of IL-1β, IL-6, and TNF-α in Shema-treated groups were decreased on the 21st day, in a certain dose-dependent manner (Figure 2C). Nevertheless, our results showed that the levels of IL-13 had no significant differences on the 11th and 21st days.

### Proteomic Analysis of Shema-Related DEPs Indicated the Therapeutic Targets of Shema Against COPD

**FIGURE 3:**
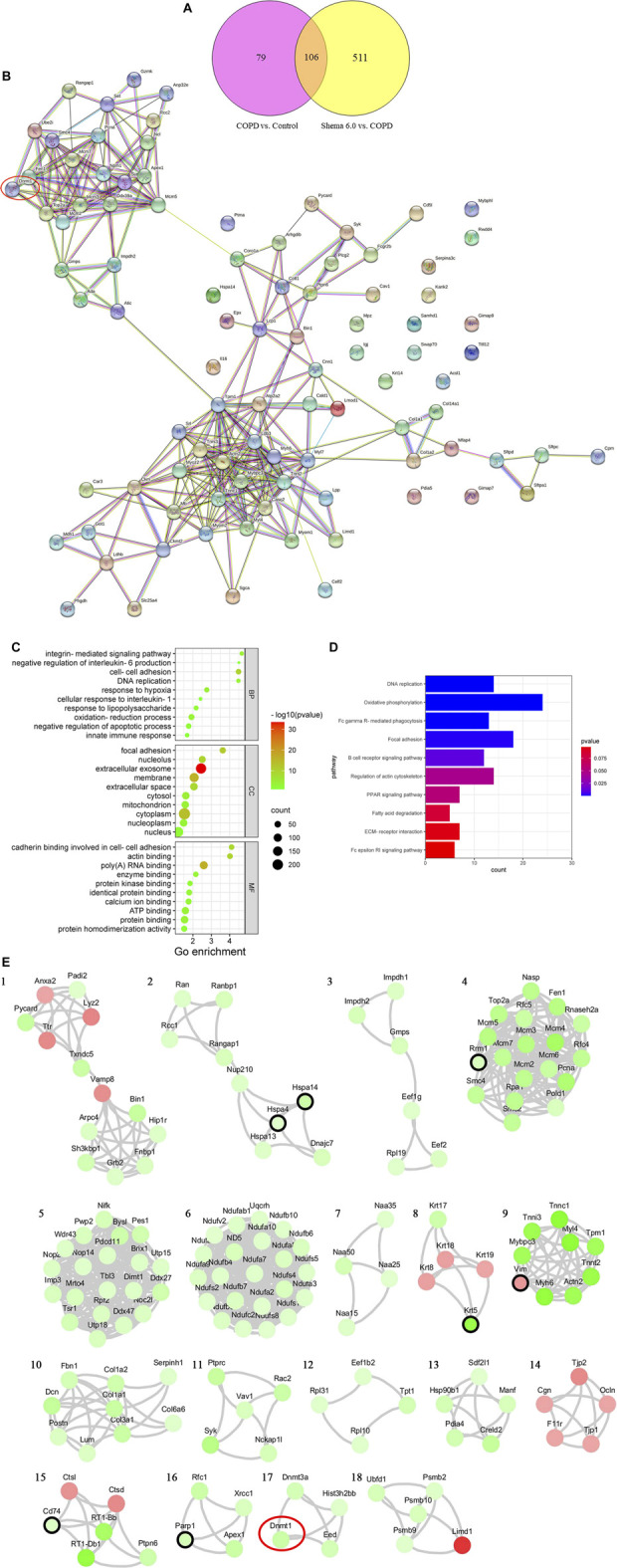
(Continued).

Using the TMT-labeled quantitative proteomic analysis, we finished high throughput screening of protein expressions, and 6,033 proteins were identified. Our results showed that 192 and 426 proteins in the Shema 6.0 group were significantly upregulated and downregulated respectively, compared with the COPD group. A total of 185 DEPs including 131 upregulated and 54 downregulated DEPs in the COPD group were identified, compared with the control group. There were 106 overlapping DEPs in these two comparisons (Figure 3A).

Subsequently, a PPI network containing 106 overlapping DEPs was constructed to further investigate the potential targets of Shema against COPD (Figure 3B). GO analysis was used to elucidate the biological significance of these DEPs on the basis of their BP, cellular component (CC), and molecular function (MF). BP category of GO analysis included oxidation-reduction process, cell-cell adhesion, negative regulation of apoptotic process, response to lipopolysaccharide, response to hypoxia, innate immune response, integrin-mediated signaling pathway, DNA replication, cellular response to interleukin-1, and negative regulation of interleukin-6 production (Figure 3C). KEGG enrichment analysis indicated that the signaling pathways of Shema against COPD were mainly related with oxidative phosphorylation, focal adhesion, DNA replication, regulation of actin cytoskeleton, Fc gamma R-mediated phagocytosis, B cell receptor signaling pathway, PPAR signaling pathway, ECM-receptor interaction, Fc epsilon RI signaling pathway, and fatty acid degradation (Figure 3D).

According to network analysis, one reliable and functional protein DNA methyltransferase 1 (DNMT1), was identified and analyzed. DNMT1 was one of the downregulated DEPs in the Shema-treated group. DNMT1 is reported to be a key maintenance methylase in mammal DNMTs family (Bayarsaihan, 2011). DNMTs-mediated aberrant methylation is linked to the pathogenesis and development of COPD (Adcock et al., 2011). DNMT1 expression in the lung tissue from COPD patients was higher than that in the lung tissue from non-smokers (Zeng et al., 2020).

We also constructed another PPI network of the DEPs by STRING database to simulate the relationships of protein interactions after Shema intervention. The protein interactions with confidence score >0.6 were reserved. Eighteen sub-clusters were extracted by the MCODE plug-in of Cytoscape software to identify highly interconnected subnetworks. The proteins in the subnetwork 2, 4, 8, 9, and 15 had the known targets of COPD, associated with chronic inflammation, pulmonary oxidation, and the imbalance of protease-antiprotease, as previously reported. The proteins in subnetwork 17 contained the direct target of Shema such as DNMT1, which was the same crucial protein contained in the PPI network of the 106 overlapping DEPs (Figure 3E). Finally, based on the above two kinds of network analysis, DNMT1 may be the important molecular target of Shema in treatment of COPD, and thus it was chosen to be verified by western blot.

### Shema Regulated the Expression of DNMT1 *via* Western Blot Validation

**FIGURE 4:**
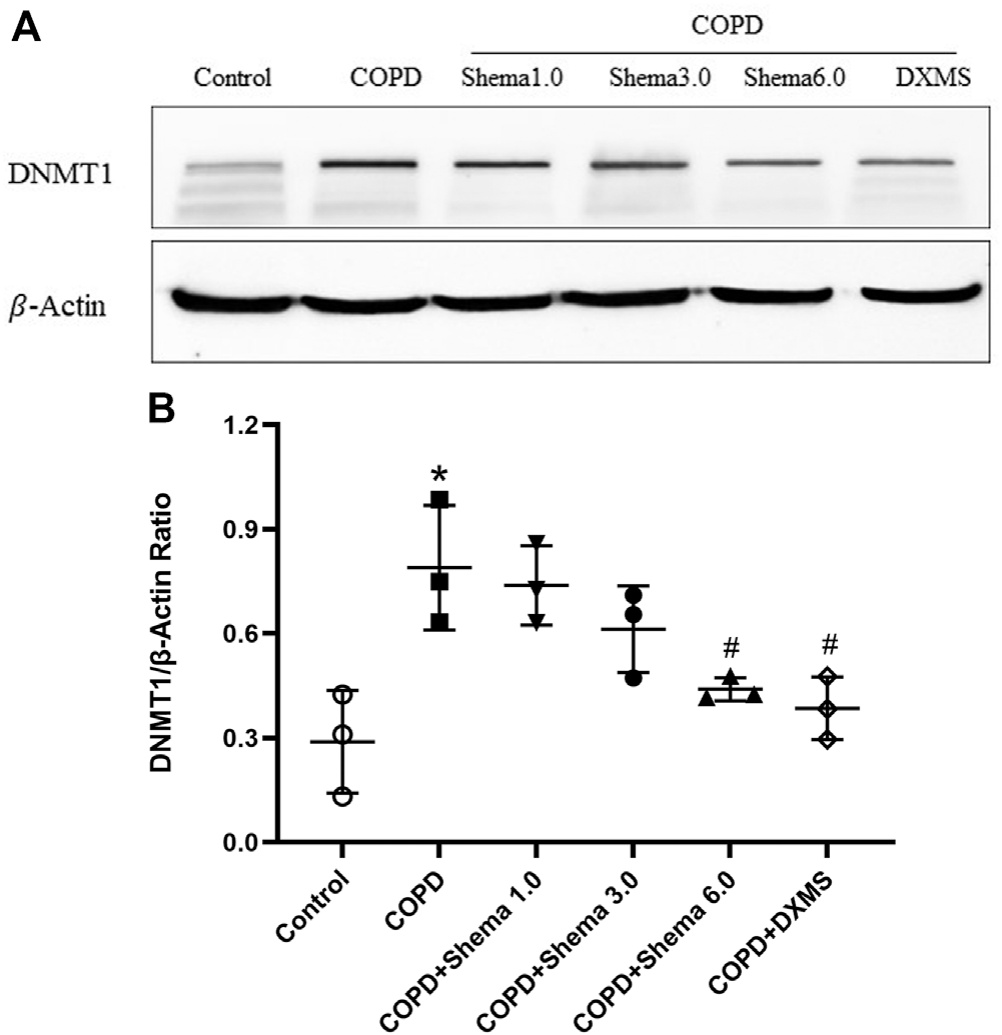
Western blot validation of DNMT1expression in the lung tissue among different groups. **(A)** Typical images of DNMT1. **(B)** Statistical quantification showing the relative expression of DNMT1. Data represented as mean ± SD, *n* = 3. *p < 0.05 vs. control group; ^#^ *p* < 0.05 vs. COPD group.

The relative content of the key functional protein DNMT1 was examined by western blot, in order to validate the expression identified by the quantitative proteomic analysis. Our data showed that the expression of DNMT1 in the COPD group was markedly upregulated compared with the control group. Additionally, compared with the COPD group, the expression of DNMT1 was significantly decreased in the Shema 6.0 group (Figure 4). The change in the expression of DNMT1 protein was consistent with the trend of the quantitative proteomic result.

## Discussion

Our research explored the protective effect and mechanism of Shema against COPD using network pharmacology. PPI network showed that inflammatory factors, such as IL-1β, IL-6, IL-13, and TNF-α, were the most critical targets contained in the pathogenesis of COPD and the therapeutic targets of Shema. GO enrichment analysis in BP category was mainly related with immune response, inflammatory reaction and defense response to bacterium. Previous studies have shown that normal immune response can prevent organs from further infection or injury during the process of inflammation. However, chronic lung inflammation such as COPD is often accompanied by immune dysfunction, leading to extensive lung damage and airway remodeling (Bhat et al., 2015). Both innate and adaptive immune systems are known to participate in the development of chronic infection in COPD (Brusselle et al., 2011).

Reports showed that IL-1-like cytokines were elevated in the lung tissue of the COPD patient, indicating the significance of an inflammasome in the pathological process of COPD (Rycroft et al., 2012). The activated pro-inflammatory caspases such as caspase 1 caused the cleavage of proinflammatory factors and the generation of IL-1β and IL-18 (Rovina et al., 2013). The serum levels of IL-6, IL-8, and TNF-α in acutely exacerbated COPD patients were increased, which may threaten pulmonary function (Lin et al., 2019). Furthermore, published studies have suggested that the release of inflammatory cytokines involving IL-1β, IL-6, and TNF-α participated in activating p38 MAPK and JNK signaling pathways in chronic lung diseases (Barnes et al., 2019). Our results showed that Shema suppressed the serum levels of inflammatory cytokines including IL-1β, IL-6, and TNF-α, which implied that Shema exerted a protective effect *via* anti-inflammation. At the same time, these data had partially verified the results concluded from network pharmacology.

Patients with COPD have declined lung functions and irreversible pulmonary limitations, as diagnosed by a ratio of FEV1/FVC <0.70 (Vestbo et al., 2013). According to the declined results of FEV0.1, FEV0.3, FVC, FEV0.1/FVC, and FEV0.3/FVC, the pulmonary ventilatory function typically dropped in COPD rats. Shema treatment significantly increased these values, indicating that Shema may improve the lung ventilative function of COPD rats. Furthermore, pathological results also revealed that Shema attenuated the infiltration of inflammatory cell, the exudation of alveolar fluid and the thickening of pulmonary interstitial, and thus protected the lung tissue from LPS-induced injury.

The proteomic profile of the lung tissue was investigated to further explore the potential targets related with Shema against COPD. A total of 106 overlapping DEPs were identified, which may be the possible therapeutic targets of Shema. Go enrichment analysis indicated that the potential regulatory mechanism of Shema treatment was mainly related with inflammation, immunity response, apoptotic process and DNA replication. Two kinds of PPI network analysis demonstrated that DNMT1 may be very important as the candidate target. Western blot result displayed that Shema markedly downregulated the expression of DNMT1 in the lung tissue of COPD rats, consistent with proteomic result.

Human DNMTs family has five members: DNMT1, DNMT2, DNMT3A, DNMT3B, and DNMT3L (Lyko, 2018). There traditional roles of DNMT1, DNMT3A, and DNMT3B are to establish and maintain DNA methylation patterns, which are highly disrupted and associated with expression changes in the small airways of COPD patients (Vucic et al., 2014). The upstream region of the Klotho promoter containing rich CpG islands is the common target for DNMTs. The Klotho protein is a transmembrane protein highly expressed in the kidney, choroid plexus, and neuron, playing a critical role in aging, inflammation, oxidative stress, calcium-phosphate metabolism, and cognitive process (Zhou et al., 2021). Recent research data have suggested that Klotho expression was decreased in blood monocytes, alveolar macrophages and respiratory epithelial cells of COPD patients or mouse models (Gao et al., 2015; Kureya et al., 2016). The expression of Klotho had an important influence on lung inflammation, and may have therapeutic implication in COPD treatment (Li et al., 2015). The treatment of cigarette smoke extract *in vitro* enhanced the levels of DNMTs (DNMT1, DNMT3A, and DNMT3B) and the methylation of Klotho promoter, ultimately leading to activate Notch signaling pathway. The inhibitions of DNMTs and Notch pathway suppressed inflammatory response and cellular apoptosis in a cell model of COPD (Qiu et al., 2018). Cigarette smoke extract treatment also promoted the expression of DNMT3A in dendritic cells of COPD mouse models, and DNMT3A regulated Th17/Treg cell balance *via* c-Jun/AIF1 axis (Huang et al., 2021). Our study further illustrated that DNMT1 was significantly increased in the lung tissue of COPD rats, which may generate novel mechanistic insight into therapeutic targets for the treatment of COPD.

**FIGURE 5:**
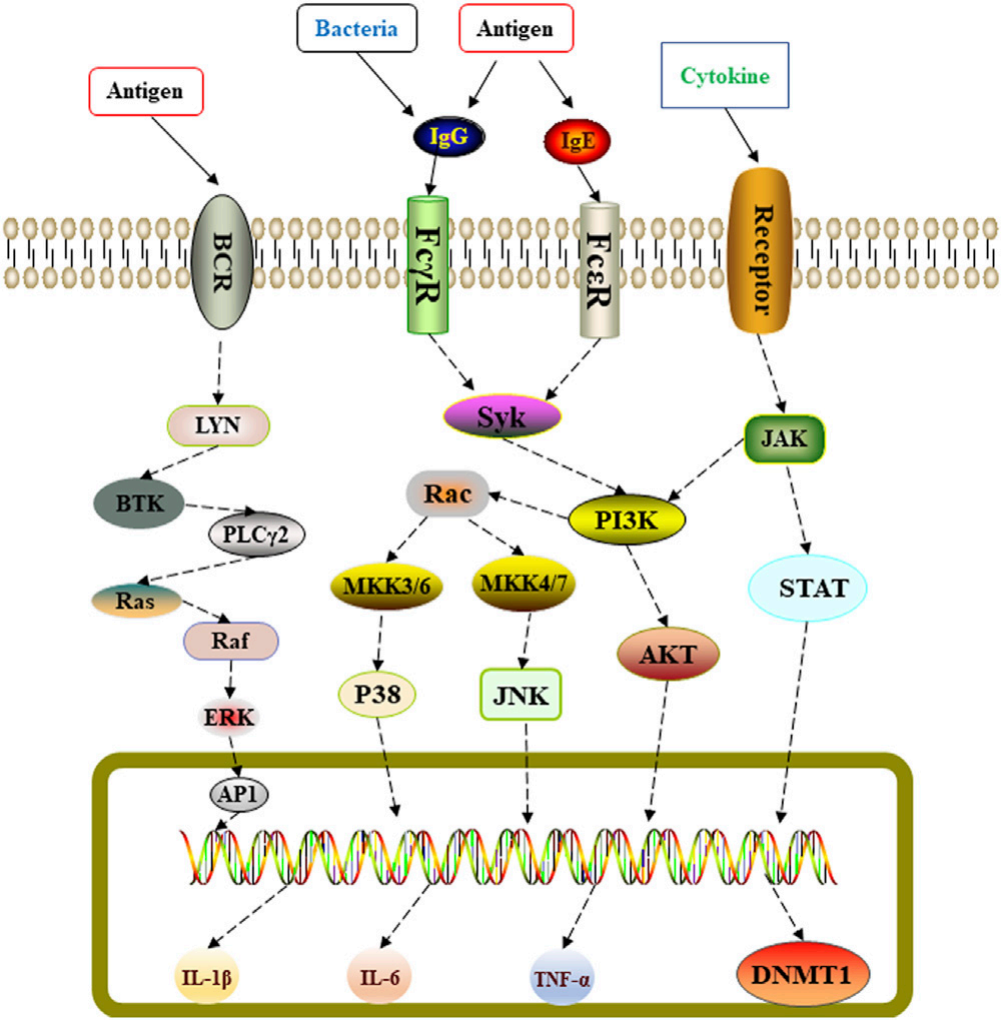
Schematic representation of Shema-mediated protective effect against LPS-induced lung injury in COPD rats.

In summary, we used network pharmacology to predict the potential molecular targets of Shema in treatment of COPD (Figure 5). Experimental research on LPS induced COPD rats proved that Shema strengthened pulmonary ventilatory function, suppressed the serum levels of inflammatory cytokines, and alleviated the pulmonary injury through regulating the expression of DNMT1. Shema mainly exerted the anti-inflammatory activity and lung-protective action involved in the protective effect on COPD. Our study shed light on the development of therapeutic strategies in treating COPD by intervening DNMT-related signaling pathways.

## Data Availability

The original contributions presented in the study are included in the article/Supplementary Material, further inquiries can be directed to the corresponding author.

## Abbreviations

COPD, chronic obstructive pulmonary disease; DNMT, DNA methyltransferase; DEPs, differentially expressed proteins; ELISA, enzyme-linked immunosorbent assay; FEV0.1, forced expiratory volume in 0.1 s; FEV0.3, forced expiratory volume in 0.3 s; FEV, forced expiratory volume; FVC, forced vital capacity; GO, gene ontology; HE, hematoxylin-eosin; IL, interleukin; KEGG, kyoto encyclopedia of genes and genomes; LPS, lipopolysaccharide; PPI, protein-protein interaction; ROS, reactive oxygen species; SD, standard deviation; Shema, Shema oral liquid; TMT, tandem mass tag; TCM, traditional Chinese medicine; TNF, tumor necrosis factor.
