# Supplementary material for: Shema Oral Liquid Ameliorates the Severity of LPS-Induced COPD via Regulating DNMT1
Source: Front Pharmacol. 2022 Jun 8;13:903593. doi: 10.3389/fphar.2022.903593 (PMC9214040; doi:10.3389/fphar.2022.903593)
Supplement: Supplementary file 1 [file Table1.docx]

**Supplementary Materials**

**Table S1. 482 putative targets of Shema oral liquid collected from BATMAN-TCM database**

| Abca1 | Cetn1 | Hrh3 | Osbpl8 | Rxrb |
| --- | --- | --- | --- | --- |
| Abcb1 | Cetn2 | Hsd17b1 | Oxer1 | Rxrg |
| Abcc2 | Cftr | Hsd17b7 | Oxt | Ryr2 |
| Abcg1 | Chrna3 | Hsp90aa1 | Oxtr | Ryr3 |
| Abhd5 | Chrnb2 | Hsp90ab1 | P2rx2 | S100a8 |
| Acaca | Chrnb4 | Htr2a | P2rx3 | S100a9 |
| Acacb | Ckb | Htr2b | P3h1 | S100g |
| Acadm | Ckm | Htr2c | P3h2 | Scgb1a1 |
| Acadsb | Ckmt1a | Htt | P3h3 | Scn10a |
| Ace | Ckmt1b | Ifng | P4ha1 | Scn1b |
| Ache | Ckmt2 | Igf1 | P4ha2 | Scn2a |
| Acsl4 | Cln3 | Il13 | P4ha3 | Scn4a |
| Acss1 | Cntnap4 | Il1b | P4hb | Scn4b |
| Acss2 | Col1a1 | Il6 | Paics | Scn5a |
| Actn2 | Colgalt1 | Ins | Parp10 | Scn8a |
| Acy1 | Colgalt2 | Insr | Pawr | Scn9a |
| Acy3 | Comt | Irx5 | Pax2 | Sdc4 |
| Adcy6 | Cplx2 | Itgav | Pax7 | Sec14l2 |
| Adh1a | Crlf1 | Itgb3 | Paxbp1 | Sec14l3 |
| Adh1b | Crtap | Iyd | Pc | Sec14l4 |
| Adh1c | Crym | Jmjd6 | Pcca | Serpinb7 |
| Adh4 | Ctsh | Kank2 | Pccb | Serpinf2 |
| Adh7 | Cxcr4 | Kcna1 | Pde10a | Shank3 |
| Adipoq | Cygb | Kcna10 | Pde11a | Shbg |
| Adk | Cyp11a1 | Kcna2 | Pde1b | Shmt1 |
| Adora1 | Cyp11b1 | Kcna3 | Pde2a | Sirt1 |
| Adora2a | Cyp11b2 | Kcna4 | Pde3b | Sirt2 |
| Adra1a | Cyp17a1 | Kcna5 | Pde4a | Slc17a7 |
| Adra1b | Cyp1a1 | Kcna6 | Pde4b | Slc18a2 |
| Adra2a | Cyp24a1 | Kcna7 | Pde4c | Slc25a12 |
| Adrb1 | Cyp27a1 | Kcnb1 | Pde4d | Slc25a13 |
| Adrb2 | Cyp27b1 | Kcnb2 | Pde5a | Slc26a6 |
| Agt | Cyp2e1 | Kcnc1 | Pde7a | Slc6a2 |
| Agtr1 | Cyp2r1 | Kcnc2 | Pde7b | Slc6a3 |
| Agtr2 | Cyp3a4 | Kcnc3 | Pde9a | Slc6a4 |
| Agtrap | Cyp4f11 | Kcnd1 | Pf4 | Slc6a8 |
| Akr1c3 | Cyp4f2 | Kcnd2 | Pgr | Snai1 |
| Akr1d1 | Dab2ip | Kcnd3 | Pkd2 | Snai2 |
| Aldh1a1 | Dars | Kcne2 | Pla2g1b | Snca |
| Aldh1a2 | Dgka | Kcne5 | Pla2r1 | Sntg2 |
| Aldh1a3 | Dgki | Kcnh2 | Plat | Snw1 |
| Aldh1b1 | Dhrs2 | Kcnip2 | Plg | Sorcs3 |
| Aldh2 | Dhrs3 | Kcnk4 | Plod1 | Sox15 |
| Aldh3a1 | Dhrs4 | Kcnma1 | Plod2 | Sparc |
| Aldh3b1 | Dhrs9 | Kcnq1 | Plod3 | Spx |
| Aldh3b2 | Dlg4 | Kif14 | Pml | Src |
| Aldh5a1 | Dlx5 | Kl | Pnp | Srd5a1 |
| Aldh8a1 | Dmtn | Lancl2 | Pnpla2 | Srd5a2 |
| Aldh9a1 | Dnaja3 | Lct | Pola1 | Stub1 |
| Alox15 | Dnmt3a | Lep | Pou1f1 | Stx1a |
| Alox15b | Dpyd | Lilrb1 | Ppara | Stx3 |
| Alox5 | Dpys | Lrat | Ppard | Suclg2 |
| Alox5ap | Drd2 | Lrrc4b | Pparg | Syt2 |
| Ampd3 | Drd5 | Magi2 | Ppp1r9b | Taar1 |
| Anapc2 | Edn1 | Maoa | Ppp2ca | Tcf3 |
| Ang | Egfr | Maob | Ppp2cb | Th |
| Ank3 | Egln1 | Mapk9 | Prdm16 | Thbd |
| Anxa1 | Egln2 | Mas1 | Prkaa1 | Thra |
| Ap3d1 | Egln3 | Mc4r | Prkaa2 | Thrb |
| Apoa1 | Esr1 | Mccc1 | Prkab1 | Tmlhe |
| Apoa2 | Esr2 | Mccc2 | Prkca | Tnf |
| Apoe | F2 | Mecom | Prkcb | Tp53 |
| Aqp8 | Fabp2 | Med1 | Prkdc | Tpo |
| Ar | Fabp3 | Metrnl | Prps1 | Trim24 |
| Arid1a | Fadd | Mip | Ptger1 | Trpa1 |
| Arrb1 | Fas | Mtap | Ptger2 | Trpm8 |
| Arx | Faslg | Mtnr1b | Ptger3 | Trpv3 |
| Ascl1 | Fbp1 | Myod1 | Ptger4 | Tspo |
| Aspa | Fgf23 | Nampt | Ptgis | Tyr |
| Asph | Fndc5 | Naprt | Ptgs1 | Tyrp1 |
| Atm | Gabra1 | Ncoa1 | Ptgs2 | Ubiad1 |
| Aurka | Gabra2 | Ncoa3 | Ptpn2 | Ubr5 |
| Avp | Gabra3 | Ndrg2 | Pygl | Uts2 |
| Avpr1a | Gamt | Neurod2 | Rab3a | Uts2r |
| Avpr2 | Gas6 | Nfib | Rab8b | Vdr |
| B4galt1 | Gata3 | Nfkb1 | Rag2 | Vkorc1 |
| Bax | Gatm | Ngfr | Rapgef2 | Vkorc1l1 |
| Bbox1 | Gc | Nkx2-1 | Rbp1 | Wls |
| Bche | Gfi1 | Nlgn1 | Rbp3 | Wnt11 |
| Bcl2 | Gja5 | Nmur2 | Rdh10 | Wnt4 |
| Bdkrb2 | Gnat1 | Nppa | Rdh11 | Zp3 |
| Bicd1 | Gnb2l1 | Nqo1 | Rdh12 | Zpr1 |
| Bmp6 | Got1 | Nr1d1 | Rdh13 |  |
| C1qtnf1 | Gpbar1 | Nr1h2 | Rdh14 |  |
| Cacna1c | Gpr143 | Nr1h3 | Rdh5 |  |
| Cacna1d | Gpr27 | Nr1h4 | Rdh8 |  |
| Cacna1g | Gpx7 | Nr1i2 | Ren |  |
| Cacna1h | Grin2a | Nr3c1 | Ret |  |
| Cacna2d1 | Grin3a | Nr3c2 | Retsat |  |
| Calb1 | Gucy1b3 | Nrxn1 | Rfk |  |
| Cat | Hacl1 | Nrxn2 | Rgcc |  |
| Cav3 | Hcn2 | Nrxn3 | Rho |  |
| Cbr1 | Hcn4 | Oca2 | Rlbp1 |  |
| Cbr3 | Hdac2 | Ogdh | Rnase1 |  |
| Ccl5 | Hdac9 | Ogfod1 | Rnase2 |  |
| Ccm2l | Heg1 | Opn4 | Rnase4 |  |
| Cd300a | Hif1a | Opn5 | Rnase8 |  |
| Cd36 | Hif1an | Oprd1 | Rs1 |  |
| Cd47 | Hpn | Oprk1 | Rxfp4 |  |
| Cdc20 | Hpx | Oprm1 | Rxra |  |

**Table S2. 69 overlapping genes between the Shema targets and COPD targets**

| Abca1 | Cftr | Hif1a | Mip | Ppara | Snai1 |
| --- | --- | --- | --- | --- | --- |
| Ace | Chrna3 | Htr2a | Nfkb1 | Pparg | Sntg2 |
| Adipoq | Chrnb4 | Ifng | Nkx2-1 | Prkcb | Src |
| Adora2a | Ckmt1b | Il13 | Nqo1 | Ptgs1 | Tnf |
| Adrb2 | Col1a1 | Il1b | Nr1i2 | Ptgs2 | Tp53 |
| Aldh2 | Cyp1a1 | Il6 | Nr3c1 | Ren | Trpa1 |
| Apoa1 | Cyp2e1 | Itgav | P2rx2 | S100a8 | Trpm8 |
| Atm | Edn1 | Kcna3 | P2rx3 | S100a9 | Vdr |
| Bcl2 | Egfr | Kl | Pde4a | Scgb1a1 | Wnt4 |
| Bicd1 | Egln2 | Lep | Pde4d | Sirt1 | Sirt2 |
| Cat | Gc | Magi2 | Pde7a | Pla2g1b | Slc6a4 |
| Ccl5 | Hdac2 | Mapk9 |  |  |  |
